# A Comparison of Teeth Measurements on Plaster and Digital Models

**DOI:** 10.3390/jcm12030943

**Published:** 2023-01-25

**Authors:** Hubert Kardach, Anna Szponar-Żurowska, Barbara Biedziak

**Affiliations:** Department of Orthodontics and Craniofacial Anomalies, Poznan University od Medical Science, 60-812 Poznan, Poland

**Keywords:** orthodontics, three-dimensional models, intraoral scanning, plaster models

## Abstract

(1) Background: Modern imaging methods and constantly developing technologies extend the range of diagnostic tools in medicine and in orthodontics. Thanks to them, scientists and doctors can use devices designed to diagnose 3D structures of the human body. The aim of the study was to assess the usefulness of digital orthodontic models as a diagnostic tool in the work of an orthodontist through a comparative analysis of the value of orthodontic measurements made on traditional plaster models and virtual models. (2) Methods: A total of 80 sets of models were made, including 40 sets of plaster models and 40 sets of digital models. A total of 48 diagnostic parameters were developed. They concerned dental parameters. (3) Results: Comparative analysis of crown height values on plaster and digital models showed statistically significant differences (*p* < 0.05) in 26 out of 48 dental parameters. (4) Conclusions: The differences between the measurements made with the software on the digital models and the measurements made with the traditional method of measurement using the digital caliper on the plaster models were small and clinically acceptable.

## 1. Introduction

Proper diagnostics is essential in planning orthodontic treatment. The correction of occlusion includes the improvement of the functional and morphological conditions of the masticatory organ, considering the patients’ expectations, which are most often related to the improvement of teeth alignment, smile aesthetics and a more attractive appearance of the face. Modern imaging methods and developing technologies extend the range of diagnostic tools in medicine as well as in orthodontics. Thanks to them, scientists and doctors can use devices designed to diagnose 3D structures of the human body. They make it possible to precisely determine the type and severity of the disorder and to plan treatment procedures through additional spatial measurements such as depth, volume or curved surface area [1].

Imaging is one of the most important diagnostic tools of orthodontists, used to assess and record the size and shape of the craniofacial structures. Nowadays, the routine techniques of two-dimensional static imaging are being replaced by three-dimensional imaging, which gives the possibility of assessing the depth of structures. 3D imaging was developed in the early 1990s and is still subject to further modifications. These technologies can be non-invasive, e.g., using magnetic resonance, ultrasound, visible light, and laser, or invasive based on X-rays. The first digital imaging technologies adopted by orthodontists included photography, pantomography, cephalometry, and periapical tissue imaging. It should be mentioned that soft and hard facial tissues and dentition are the three main elements, also called triads, analyzed in detail in orthodontics and orthognathic surgery [2]. Therefore, imaging those structures is one of the important diagnostic tools for clinicians in making decisions about the treatment method [3].

Orthodontic models are an indispensable diagnostic tool for an orthodontist. Thanks to them, it becomes possible to carry out a thorough analysis of the size, shape and position of the teeth, the shape of the dental arches, and to determine the mutual relations between them. In addition, individual orthodontic appliances or their individual components are made in the laboratory on orthodontic models [4]. Until recently, orthodontic plaster models were the only available three-dimensional information carrier that accurately reflected the patient’s occlusal situation.

Due to the development of computer techniques and the introduction of oral tissue scanning methods, it became possible to create three-dimensional digital orthodontic models [5]. There are two methods of creating a virtual model. The first one is the direct technique, where during the examination of the patient with the help of specialized visualization techniques, e.g., with the use of computer tomography, magnetic resonance, or in the process of intraoral scanning with the use of an intraoral scanner, records of the oral cavity conditions are made in the form of a file. In order to obtain a complete picture of the dental arch, it is necessary to scan five areas: the buccal, occlusal and lingual surfaces, as well as the proximal and distal interdental surfaces [6]. The second option is indirect scanning, which consists of scanning a previously made plaster model in a traditional way after taking impressions in the patient’s mouth using the impression material or scanning the impressions themselves. This method uses non-contact optical scanning processes using a laser, white light, or piezoelectric tactile light. There are also destructive techniques consisting of analyzing the solid plaster model by revealing its successive sections by shearing layers of a predetermined thickness with simultaneous recording. Layers are imaged with a scanner, and a digital record of the model is generated using specialized computer software [7]. Three-dimensional digital impressions were first introduced in 1987 by CEREC 1 (Siemens, Munich, Germany) using an infrared camera and optical powder distributed over the tooth surface. Over the years, the development of computer hardware and software has radically improved the technologies, making them much faster and simpler, replacing traditional impressions with the use of impression material in many dental and orthodontic offices. Computer-aided design and manufacturing (CAD/CAM) systems are based on three basic steps: (1) data collection and digitization, (2) data processing and design, and (3) production. The development of digital impressions made it possible to create high-resolution virtual 3D models [8,9]. Mapping the structures of the teeth and surrounding tissues is carried out using non-contact optical technologies. Thanks to this, negative aspects of traditional impressions, such as discomfort for the patient, inaccuracy or work in the laboratory, are eliminated. Although plaster models have been used for years as routine dental records for analyzing dentition, they nonetheless have several disadvantages. They are time-consuming, space-consuming to store, brittle, degrading and can be damaged when handled. Thus, digital models are an alternative to traditional plaster models. Their advantages in orthodontic diagnosis and treatment planning include easy and fast electronic data transfer, immediate access, and reduced space requirements [10]. The emergence of new technological solutions has become the subject of research on the compliance and reliability of measurements of plaster models and digital models. The first studies concerned the analysis of digital models obtained by scanning plaster models with a 3D model scanner. Most researchers have shown that a digital model scanned with a 3D model scanner shows high accuracy compared to conventional plaster models [11,12,13,14]. Some researchers reported that digital models provide more accurate imaging than the plaster model [15].

### Purpose of Research

The aim of the study was to assess the usefulness of digital orthodontic models as a diagnostic tool in the work of an orthodontist through a comparative analysis of the values of dental measurements, including the width and height of the clinical crown, made on traditional plaster models and virtual models.

## 2. Materials and Methods

The study group consisted of 40 patients. A total of 80 sets of models were made, including 40 sets of plaster models and 40 sets of digital models. The age range of the study group ranged from 16 to 37 years. There were 24 women and 16 men among the respondents.

**Figure 1:**
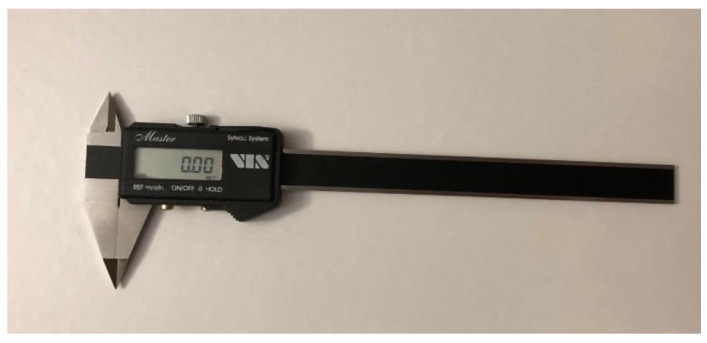
Electronic caliper.

**Figure 2:**
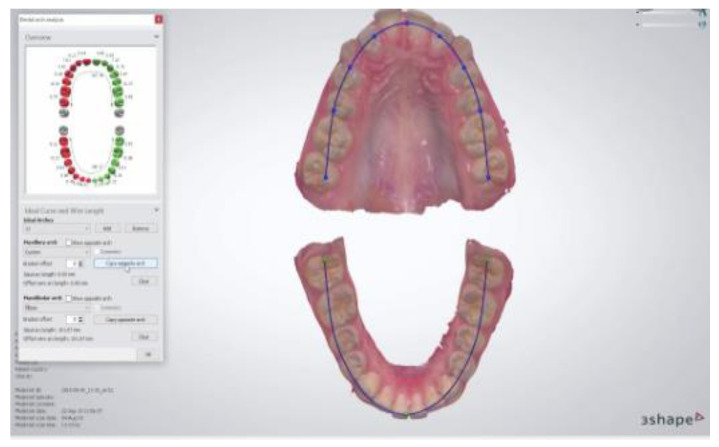
OrthoAnalyzer.

Each patient underwent a medical interview and clinical dental examination. Impressions for plaster models were taken, and an intraoral scan was performed. Taking alginate impressions and making digital scans of every patient took place on the same day. Anatomical impressions were taken in all patients using one type of impression material. An alginate mass with the brand name Kromopan was used for this purpose. Each new packaging of alginate mass, before opening, was checked for the tightness of the bag closure and the expiry date. The impression was taken with a disposable orthodontic spoon of an appropriate size for the patient. Factory-made perforations of the impression trays additionally increased the retention of the impression material. In the next stage, the impressions were secured and transported in a humid environment to the prosthetic laboratory as soon as possible, not exceeding 2 h. Next, the model was cast using Type III prosthetic plaster, characterized by a perfect white color and with high strength and low expansion. During the same visit, an intraoral scan was performed using the 3Shape Intraoral Scan TRIOS3 device. In order for the procedure to be performed correctly, the surfaces of the teeth were initially dried with compressed air, and then the patient’s teeth were scanned with a sterile scanner head, starting from the right quadrant in the maxilla. Thanks to the performed activities, virtual patient models were obtained, which were archived in the 3Shape server. The basic research method used in the study was orthodontic measurements. In the case of diagnostic plaster models, measurements were made using a manual electronic caliper by FWP VIS SA model MAUa Basic, an optimum master with a measuring range of 150 mm, with an accuracy of 0.01 mm (Figure 1). In order to minimize the occurrence of a measurement error, the same measurement conditions were created for the lighting in the room and the temperature present in it. To determine the compliance of the performed measurements, 10 parameters were selected, 5 of which concerned dental measurements and 5 of which concerned linear measurements. The orthodontic analysis of digital models was performed using a digital caliper using the OrthoAnalyzer 3Shape software, also with an accuracy of 0.01 mm (Figure 2).

**Figure 3:**
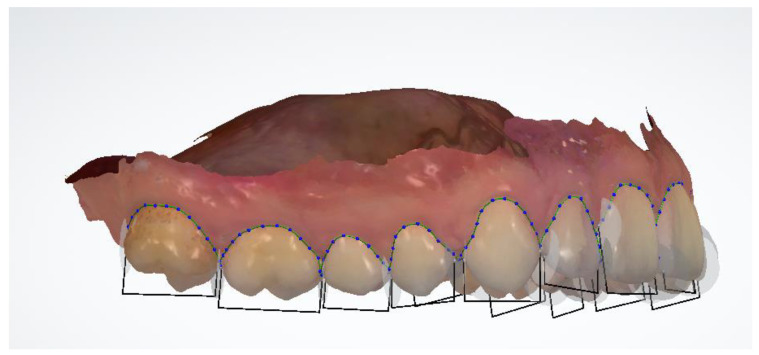
Mesial-distal width, OrthoAnalyzer.

**Figure 4:**
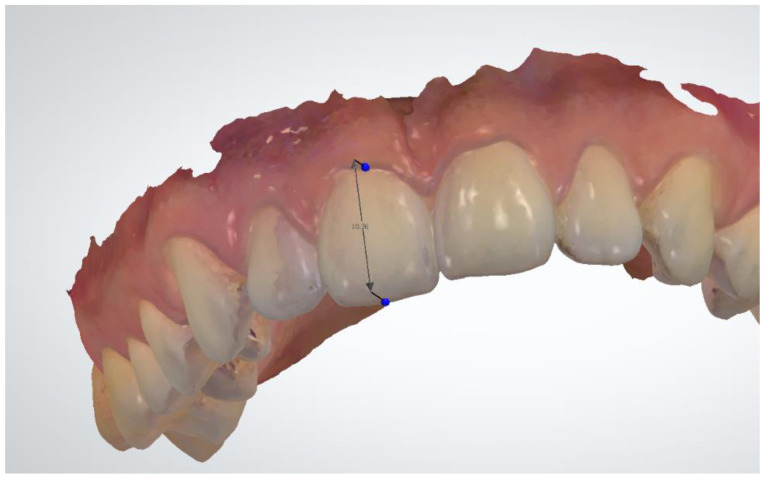
Hight measurement, OrthoAnalyzer.

The first 24 parameters (1–24) concerned the measurements of the width of clinical crowns of individual teeth. A total of 24 teeth were measured, starting from the first molar in the upper right jaw and measuring the teeth successively to the first molar in the left jaw. In the mandible, however, the measurement started from the first molar on the left side, ending with the first molar on the right side. The mesial-distal width of each tooth was measured at the widest distance between the contact points (Figure 3). On the incisors and canines, the measurement was made on the labial surface (Figure 4). On premolars and first molars, the measurement was performed in the occlusal plane. The caliper arms were set parallel to the long axis of the tooth. In the case of analysis performed on digital models using the OrthoAnalyzer 3Shape software, the above-mentioned points were marked on a virtual model and connected in a straight line by measuring the resulting section.

The next parameters (25–48) were measurements of the height of the clinical crown of individual teeth. In all tooth groups, they were made on the labial or buccal surfaces. In the case of incisors, measurements were taken in the center of the crown along the long axis of the tooth from the gingival line to the incisal edge. On the canines and premolars, measurements were taken from the gingival line to the top of the cusps. Measurements of the height of the molar crowns in both the maxilla and the mandible were modified due to the anatomical structure. Measurements were taken in the long axis of the molars in the buccal groove from the gum line to the occlusal surface.

## 3. Results

A comparison of the crown width values on analog and digital models showed statistically significant differences in the measurements of ten teeth: 16, 21, 22, 23, 32, 33, 34, 43, 45, 46 (Table 1 and Table 2).

Higher values of teeth width measurements in digital models were observed in the area of the posterior teeth on the right side of the upper and lower dental arches (16, 45, 46). On the other hand, smaller values of the teeth width measurements in digital models were observed in the area of the front teeth (21, 22, 23, 32, 33, 34, 43). The largest differences in the measurement values concerned the width of teeth 23 and 16. For tooth 23, the average measurement in the digital model was, on average, 0.30 mm smaller than that of the plaster model, while for tooth 16, the average measurement in the digital model was, on average, 0.23 mm larger than the measurement in a plaster model.

Comparative analysis of crown height values on plaster and digital models showed statistically significant differences (*p* < 0.05) in the measurements of sixteen teeth: 16, 13, 12, 11, 21, 22, 23, 25, 26, 31, 32, 36, 41, 42, 45, 46 (Table 3 and Table 4). Lower values of the measurements of the height of the clinical crown of the teeth on digital models were observed in the area of the posterior teeth in the upper and lower dental arches on the left and right sides (16, 25, 26, 36, 45, 46). The remaining eleven statistically significant differences were related to the increased value of the crown height measurements on digital models. These changes concerned only the front teeth, both in the upper and lower arch (13, 12, 11, 21, 22, 23, 31, 32, 41, 42). The differences in the mean values of the measurements ranged from 0.08 to 0.40 mm. The greatest changes concerned the measurements of teeth 11, 21, 26, and 46. For tooth 46, the average measurement on the digital model was 0.40 mm lower than the measurement on the plaster model. The mean clinical height dimension of tooth 26 was smaller by 0.35 mm in the digital model than in the plaster model. For teeth 11 and 21, the average measurements of crown height on digital models were 0.33 mm and 0.35 mm higher, respectively, than in plaster models.

## 4. Discussion

Correct diagnosis and treatment plans are the key to therapeutic success in orthodontics. Dental or orthodontic indicators have always been the main element of the initial patient examination. Measurements of teeth and dental arches are particularly important in the analysis of patients with tooth crown-size disharmony. Their conduct is also necessary in borderline cases when choosing a method of non-extraction or tooth extraction. Watanabe-Kanno et al., Leifert et al., Zilberman et al., and Asquith et al. analyzed from 10 to 25 diagnostic models [12,16,17,18]. Based on the results of these studies, in this study, it was decided to carry out the analysis on 40 sets of diagnostic models. In the present author’s research, it was observed that the significant differences in dental measurements in plaster and digital models concerned 30% of the measured teeth. Out of 48 dental measurements, differences were noted for 16 measurements. They were small and ranged between 0.08 and 0.40 mm. Similar results were obtained by Aly P. et al., who investigated the mesial-distal teeth in plaster models, digital models obtained by scanning, and models produced in the 3D printing process. The differences observed by the authors ranged from 0.016 to 0.142 [19]. 

Hunter and Priest, in 1960, described their method of measuring the mesial-distal width [20], which was used in their own study. Comparisons of the width of clinical crowns showed statistically significant differences for 10 teeth, while the measurements of the width of the posterior teeth on the digital models were greater than those on the plaster models, and the opposite was true in the anterior segment: the measurements of the width of the crowns on the digital models were smaller than those on 86 plaster models. Santoro et al. conducted a comparative analysis of the width of clinical crowns of teeth on plaster models and their digital scans. The differences in the width of the teeth reached 0.38 mm. They observed a reduction in the value of the width of the teeth on the scanned models [14,21]. Additionally, Jedlińska, in her research, confirmed the consistency of the measurements of the width of the crowns on the plaster models and their scans [5]. Cuperes et al., in their examinations using an intraoral scanner Lava Chairside Oral Scanner, found that the teeth width measurements made in digital models were larger [22].

In our own research, both lower and higher measurement values were found in the average width of the teeth in the maxilla and the mandible in digital models. According to the available literature, the differences in measurements may result from the fact that the impressions shrink during transport to the prosthetic laboratory. Despite the fact that in our study, impressions were cast within 2 h of their taking, according to Coleman et al., deformation changes in impressions may occur despite their short storage and quick casting [23]. Alcan et al. report that the mean absolute measurement error between the measurements made on the reference model and the measurements made on the plaster models taken within 1 h of collection is 1.285%. In the same studies, the comparison of the values of the measurements made on the reference model and digital models was only 0.695% [24]. Differences in measurements on plaster and digital models may also be caused by a low precision of restoration on proximal surfaces, which makes the positioning of the approximate measurement points difficult. Another reason may be the increased accuracy of virtual techniques. Accurately positioning a thin and small cursor anywhere in the tooth being measured is much easier than using the oversized caliper gauge used for measurements on plaster models. The main limitation of the digital method is the identification of the measuring points rather than the use of a scanning device or type of software. Dalestra et al. point out that, especially in the case of digital measurements, the so-called learning curve and the experience of the dentist performing the measurement are important. In their research, they shared their observations that despite the perfect training and calibration of two examiners, the first of them was not able to perfectly recreate the landmarks chosen by the second person. On the other hand, Santoro et al. and Rossini et al. noticed that differences in the measurements of teeth using traditional and digital techniques may be caused by morphological changes in the teeth [14,24]. According to Asquith and McIntyre, systemic errors exceeding 0.5 mm in the measurements of single teeth or specific sections, or when the measurement error is above 5%, are clinically unacceptable [25]. On the other hand, Naidu et al., Santoro et al., and Reuschl et al. claim that a measurement error of 0.5 mm or even slightly greater does not seem to be of clinical significance [21,26,27].

Mesial-distal measurements of individual teeth are used to calculate the value of the Bolton index, which is an important factor in making a treatment decision. Additionally, although in the case of measurements of single teeth, these differences do not seem significant, after their summation and determination of the Bolton index, they may turn out to be clinically significant. Sherrard et al. stated in their studies on the reliability of the Bolton index analysis that clinically significant measurement errors (>1.5 mm) may occur with crowding of 3 mm [28].

In research conducted by Naidu et at., Reushl et al., and Wiranto et al., the difference in measurements using hand calipers or virtual analyzes was clinically insignificant and was less than 1.5 mm in these studies [26,27,29]. Therefore, it should be emphasized that the differences in the measurement values are small and do not change the therapeutic diagnosis. Additionally, the therapeutic and treatment decisions made do not differ significantly in the case of calculations of orthodontic indicators on digital models or on plaster models [30].

It is noticeable in the literature that the topic of comparing the results obtained from plaster and virtual models is still popular and often published. All authors emphasize that intraoral scanners and virtual models can replace the classic ones. Moreover, the scanning procedure itself takes less time at the dentist’s chair and is less unpleasant for the patient than a standard dental impression. It was also published that patients, when asked which method of taking impressions was more satisfactory for them, definitely chose virtual techniques. An important fact is noted by Solabrietta et al., who claim that these two techniques should not be compared because facilitating the dentist’s daily work and improving the patient’s comfort should always be of the highest priority [31,32]. 

It should be mentioned that intraoral scanners and the obtained virtual impressions have streamlined and improved the quality of some modern techniques of orthodontic treatment. Using the width of the teeth is important during treatment, for example, with the Invisalign method. The interest of patients in this method of treatment, due to the wearing of dental trays instead of orthodontic brackets, is constantly growing. Determining the width of the teeth on the virtual models helps in starting the orthodontic stripping treatment and setting the correct dental arch in the program [33].

These small differences in measurement values, while not affecting the type of treatment method, may nevertheless have an effect on the range of action, as in the thickness of the ground contact surfaces of the teeth. An analysis of the space requirements for aligning the teeth by these two techniques can translate into an assessment of the extent of the required reduction in the width of clinical crowns.

## 5. Conclusions

A significant agreement was observed between the measurements of the size of the teeth made on digital and plaster models. The differences between the measurements made with the software on the digital models compared to the measurements made with the traditional method of measurement using the digital caliper on the plaster models were small and clinically acceptable.

Digital models constitute a new generation of precise diagnostic tools in orthodontics, which, thanks to the ease of copying and sending, opens up new possibilities for consultation and multi-specialist planning with various clinical centers. This study shows that the traditional method of producing orthodontic plaster models and the method of producing digital models using intraoral scanning can be used interchangeably.

## Figures and Tables

**Table 1:** Comparison of the mean values of the tooth width (MD) measurements in the upper arch.

	TOOTH											
Mean Measured Value MD (Mean)	16	15	14	13	12	11	21	22	23	24	25	26
Plaster	10.28	6.43	6.68	7.70	6.49	8.25	8.19	6.46	7.65	6.58	6.51	9.93
Digital	10.51	6.41	6.76	7.60	6.44	8.31	8.08	6.28	7.35	6.53	6.45	9.95
Average difference	−0.23	0.02	−0.08	0.1	0.05	−0.06	0.11	0.18	0.30	0.05	0.05	−0.02
*p*	0.0010	0.7049	0.1325	0.0939	0.1726	0.1243	0.0043	0.0000	0.0000	0.3720	0.3015	0.7965

**Table 2:** Comparison of the mean values of the tooth width (MD) measurements in the lower arch.

	TOOTH											
Mean Measured Value MD (Mean)	36	35	34	33	32	31	41	42	43	44	45	46
Plaster	10.32	6.87	6.92	6.52	5.77	5.21	5.17	5.74	6.52	6.98	6.87	10.43
Digital	10.40	6.90	6.71	6.36	5.62	5.18	5.20	5.77	6.38	6.99	6.98	10.59
Average difference	−0.08	−0.03	0.21	0.16	0.15	0.03	−0.03	−0.03	0.14	−0.01	−0.11	−0.16
*p*	0.1186	0.4687	0.0000	0.0170	0.0003	0.3395	0.3252	0.4631	0.0246	0.8191	0.0169	0.0044

**Table 3:** Comparison of the mean values of the measurements of the height of the clinical crown (H) in the upper arch.

	TOOTH											
Average Value of the H Measurement (Mean)	16	15	14	13	12	11	21	22	23	24	25	26
Plaster	5.60	6.60	7.75	9.23	8.23	9.55	9.61	8.36	9.39	7.72	6.42	5.50
Digital	5.43	6.57	7.76	9.44	8.50	9.87	9.95	8.65	9.60	7.70	6.34	5.15
Average difference	0.17	0.03	−0.01	−0.21	−0.27	−0.32	−0.34	−0.29	−0.21	0.02	0.08	0.35
*p*	0.0108	0.4736	0.8313	0.0001	0.0000	0.0000	0.0000	0.0000	0.0000	0.6675	0.0158	0.0000

**Table 4:** Comparison of the mean values of the measurements of the height of the clinical crown (H) in the lower arch.

	TOOTH											
Average Value of the H Measurement (Mean)	36	35	34	33	32	31	41	42	43	44	45	46
Plaster	6.22	7.17	8.35	9.49	8.47	7.94	8.26	8.27	9.51	8.07	7.14	6.28
Digital	6.01	7.11	8.35	9.52	8.65	8.04	8.38	8.40	9.47	8.07	6.97	5.88
Average difference	0.21	0.06	0.0	−0.03	−0.18	−0.10	−0.12	−0.13	0.04	0.00	0.17	0.40
*p*	0.0004	0.1049	0.9440	0.5413	0.0004	0.0282	0.0018	0.0004	0.3948	0.9200	0.0015	0.0000

## Data Availability

The study was not publicly funded. Data are not open-ended.
